# Deep Neural Network-Based Video Processing to Obtain Dual-Task Upper-Extremity Motor Performance Toward Assessment of Cognitive and Motor Function

**DOI:** 10.1109/TNSRE.2022.3228073

**Published:** 2023-02-01

**Authors:** Zilong Liu, Changhong Wang, Guanzheng Liu, Bijan Najafi

**Affiliations:** School of Biomedical Engineering, Sun Yat-Sen University, Shenzhen Campus, Shenzhen, Guangdong 518000, China.; Michael E. DeBakey Department of Surgery, the Baylor College of Medicine, Houston, TX 77030 USA.; School of Biomedical Engineering, Shenzhen Campus of Sun Yat-Sen University, Shenzhen, Guangdong 518000, China; School of Biomedical Engineering, Sun Yat-Sen University, Shenzhen Campus, Shenzhen, Guangdong 518000, China.; Michael E. DeBakey Department of Surgery, Baylor College of Medicine, Houston, TX 77030 USA; BioSensics LLC., Newton, MA 02458 USA

**Keywords:** Dementia, motoric cognitive risk syndrome, telehealth, tele-medicine, deep residual neural network, mobile health, video processing

## Abstract

Dementia is an increasing global health challenge. Motoric Cognitive Risk Syndrome (MCR) is a predementia stage that can be used to predict future occurrence of dementia. Traditionally, gait speed and subjective memory complaints are used to identify older adults with MCR. Our previous studies indicated that dual-task upper-extremity motor performance (DTUEMP) quantified by a single wrist-worn sensor was correlated with both motor and cognitive function. Therefore, the DTUEMP had a potential to be used in the diagnosis of MCR. Instead of using inertial sensors to capture kinematic data of upper-extremity movements, here we proposed a deep neural network-based video processing model to obtain DTUEMP metrics from a 20-second repetitive elbow flexion-extension test under dual-task condition. In details, we used a deep residual neural network to obtain joint coordinate set of the elbow and wrist in each frame, and then used optical flow method to correct the joint coordinates generated by the neural network. The coordinate sets of all frames in a video recording were used to generate an angle sequence which represents rotation angle of the line between the wrist and elbow. Then, the DTUEMP metrics (the mean and SD of flexion and extension phase) were derived from angle sequences. Multi-task learning (MTL) was used to assess cognitive and motor function represented by MMSE and TUG scores based on DTUEMP metrics, with single-task learning (STL) linear model as a benchmark. The results showed a good agreement (r ≥ 0.80 and ICC ≥ 0.58) between the derived DTUEMP metrics from our proposed model and the ones from clinically validated sensor processing model. We also found that there were correlations with statistical significance (p < 0.05) between some of video-derived DTUEMP metrics (i.e. the mean of flexion time and extension time) and clinical cognitive scale (Mini-Mental State Examination, MMSE). Additionally, some of video-derived DTUEMP metrics (i.e. the mean and standard deviation of flexion time and extension time) were also associated with the scores of timed-up and go (TUG) which is a gold standard to measure functional mobility. Mean absolute percentage error (MAPE) of MTL surpassed that of STL (For MMSE, MTL: 18.63%, STL: 23.18%. For TUG, MTL: 17.88%, STL: 22.53%). The experiments with different light conditions and shot angles verified the robustness of our proposed video processing model to extract DTUEMP metrics in potentially various home environments (r ≥ 0.58 and ICC ≥ 0.71). This study shows possibility of replacing sensor processing model with video processing model for analyzing the DTUEMP and a promising future to adjuvant diagnosis of MCR via a mobile platform.

## I. Introduction

THE aging of world population leads to the shortage of medical resources for the older adults. Care support of the older adults with dementia has brought a heavy burden on many families [1]. The identification of preclinical stages of dementia is important for dementia prevention [2]. Traditionally, identification of preclinical stages of dementia is based on neuropsychological test, blood biomarkers and neuroimaging [3]. However, these methods often require a large number of medical resources and high economic costs, thus they aren’t suitable for low-income regions and countries. Therefore, a quick, objective and reliable assessment tool which can be used in remote and low-income regions is beneficial to popularize predementia examination for the older adults and reduce the burden of medical resources.

Motoric Cognitive Risk Syndrome (MCR) is a preclinical stage for dementia which is defined as the presence of slow gait speed and subjective memory complaints without cognitive or functional impairment [4]. MCR is associated with increased incidence of dementia, particularly Alzheimer’s Disease (AD) and Vascular Dementia [3], [4]. Additionally, MCR increases the risk of other geriatric outcomes including frailty, disability, falls and mortality [4], [5]. The prevalence of MCR was 9.7% in a pooled analysis of 26,802 older adults from 17 countries in 2014 [3]. MCR can be identified by a gait assessment, a cognitive complaints questionnaire, and a functional inquiry which can be performed by clinicians in the outpatient clinic [6]. However, in remote and low-income regions, the older adults seldom take the initiative to go to the clinic to check their cognitive status because of inconvenience and medical expense. Without proper preventive measures (e.g., physical activity [7]), older adults with MCR are likely to develop major cognitive decline and dementia. Therefore, it is necessary to develop an assessment tool of MCR to screen older adults with high risk of cognitive decline and to recommend such cohort to receive a full neuropsychologic evaluation by primary care physician. Then, a series of preventive measures will be performed to delay the occurrence of dementia, including maintaining systolic blood pressure of 130 mm Hg, encouraging use of hearing aids, reducing smoke, limitation of alcohol use, reducing obesity and other lifestyle interventions. Modifying these risk factors might prevent or delay up to 40% of dementias [2].

The dual-task motor paradigm (e.g., dual-task walking) is a method for assessing executive function and attention performance which can be used for quantifying both cognitive function and motor capacity [8], [9], [10], [11], [12]. Poor dual-task motor performance is associated with cognitive impairment [8], [13]. Therefore, dual-task motor performance test can be used as an additional diagnosis tool of MCR. In previous studies, researchers used an inertial sensor to capture the movement of the upper-extremity during the trial [12]. However, the extra purchase of sensors (a wrist-worn sensor is worth at least $200) is unacceptable to many of the older adults in low-income regions. Meanwhile the wearing and use of sensors are not friendly to the older adults. In details, during the trial the participant needs to wear the inertial sensor on the wrist in a certain calibrated orientation. The process of taking on the sensor and human-computer interaction of data collection software on a tablet normally require a professional tester involving in the trial. To address these barriers, we develop a new video processing model to analyze dual-task upper-extremity motor performance (DTUEMP) which can be conveniently integrated into a smartphone with a camera module, thereby reducing the cost of purchasing a specific data acquisition equipment, such as wearable inertial sensors. Our previous study used a color segmentation algorithm to track the wrist coordinates during the test [14]. This method requires the older adults to tie a cloth belt of specific color to their wrist for coordinate tracking [14], which is not very convenient and is greatly affected by the light and the color of the older people’s garment, so in this paper we proposed a video processing model based on a residual neural network and optical flow method to automatically track movements of upper extremity, without relying an additional specific colored cloth belt on the wrist.

In 2014, K. He et al. proposed a residual framework to ease training the network that are substantially deeper than those used previously. This framework has already been widely used in pose recognition [15], [16]. Because of deep residual networks’ potential in pose recognition, we proposed a video processing model based on a deep residual neural network to obtain DTUEMP from an elbow flexion-extension test under dual-task conditions. In the elbow flexion-extension test under dual-task condition, participants need to count down from a random two-digit number by one while doing elbow flexion and extension. We used this deep neural network to obtain the coordinate set of the wrist and elbow during the trial. To improve the recognition accuracy of the neural network, the optical flow method was used to correct the wrong coordinate set of the neural network in some frames [17]. After correction, the final output coordinate set was used to generate angle sequence. Then, DTUEMP metrics were extracted from the angle sequence. We verified the effectiveness of our proposed model by testing the correlation between metrics derived from videos and sensors. We also analyzed the correlation between the DTUEMP metrics derived from our proposed video processing model and clinical cognitive scale (Mini-Mental State Examination, MMSE) as well as motor function index (timed up and go (TUG) scores) [18], [19]. We designed an experiment to verify robustness of our proposed model transplanted to mobile phones, in which the participants performed elbow flexion and extension test under different light conditions and shot angles. Lastly, both multi-task learning (MTL) and single-task learning (STL) models were used to perform regression between DTUEMP metrics and MMSE as well as TUG scores with five-fold cross validation [20]. We used mean absolute percentage error (MAPE) to measure the performance of STL and MTL model.

## II. Method

### A. The Elbow Flexion-Extension Test

During the test, participants were asked to perform a dual-task upper-extremity functional test: as a motor task, they repetitively flexed and extended their dominant elbow to full flexion and extension position as quickly as possible for 20 seconds. At the same time, the participants conducted a cognitive task: counting backward by one from random two-digit number [21]. During the trial, a camera (Vixia HF R800 Video Camera, Canon U.S.A. Inc., Melville, NY) was held in the tester’s hand and focused on the participant’s right-hand side to capture the upper-extremity movements. The natural tiny swinging of the tester’s arm may introduce possible unsteadiness and blur in the video recording to challenge our proposed video processing model, simulating a real telehealth scenario in which the older adult’s family member holds the smartphone in his hand to take video recording. The video recordings do not contain any facial information by manual control of viewing angles. The video frame rate is 30 frame per seconds (FPS).

### B. Participants

Older adults (age 65 year or older) with either mild cognitive impairment, mild dementia, or Alzheimer’s disease diagnosed by certificated neurologists were recruited from the Geriatrics Clinic and Alzheimer’s Disease and Memory Disorder Center at Baylor College of Medicine. Participants were excluded from the study if they were non-ambulatory or had a severe gait impairment (e.g., unable to walk 10-meter independently with or without an assistive device); had other neurological diseases associated with cognitive impairment (stroke, etc.); had any clinically significant medical or psychiatric condition; had severe visual and/or hearing impairment; with changes in psychotropic medications in the last 6 weeks. All participants signed a consent form for this study.

### C. Video Processing Model Based on Deep Neural Network

Our video processing model consists of two parts. The first part is pose recognition based on a deep neural network by B. Xiao et al. [16], using the video of upper-extremity motion as input. The neural network outputs a coordinate set of the participant’s elbow and wrist extracted from all frames of the video. The coordinate set is used to generate angle sequence *A*^*N*^ which represents rotation angle of the line between the wrist and elbow. The second part is a coordinate-correction method based on optical flow to correct possibly wrong coordinates outputted from the pose recognition algorithm when processing some frames of the video.

**Fig. 1.**
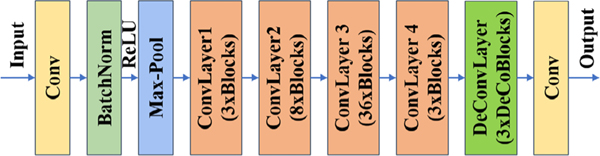
The structure of deep neural network for pose recognition.

Our pose recognition approach uses a residual neural network with a depth of 152 layers. It is a common backbone network for image feature extraction and pose estimation [22]. Our neural network adds some deconvolutional blocks over the last convolutional layer [15], [16]. The network structure is shown in Fig. 1. The deconvolution part consists of three deconvolution blocks which are composed of a deconvolutional layer with batch normalization and rectified linear Units (ReLU) activation [16], [23]. Each deconvolutional layer has 256 filters with 4 × 4 kernel [16] and the stride is 2. A 1 ×1 convolutional layer is added at last to generate predicted heatmaps [16]. Coordinates are obtained from heatmaps by the local maximum method. The network is trained on the COCO *train2017* dataset (includes 57K images and 150K person instances) [24].

**Fig. 2.**
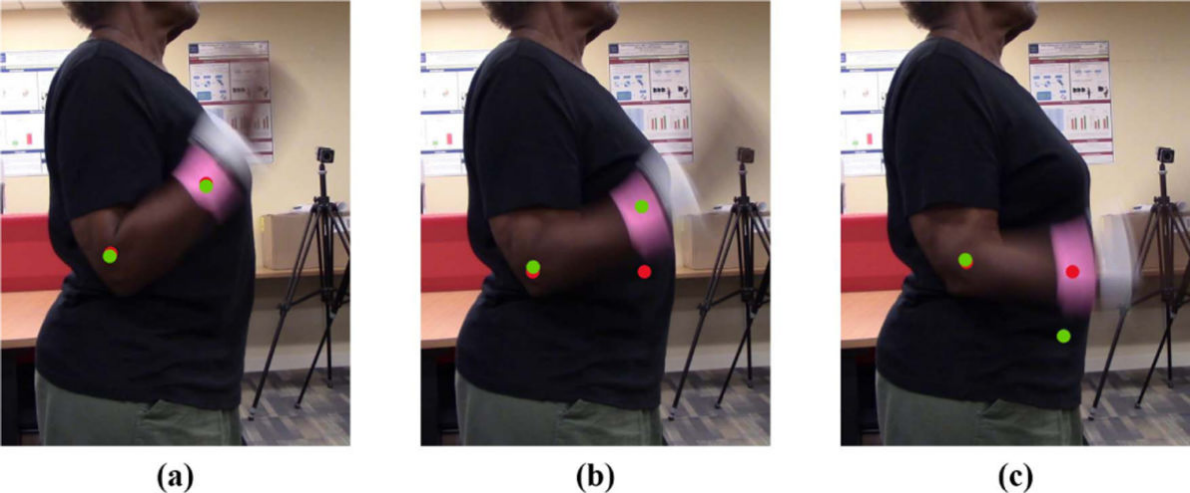
Some example frames for the elbow flexion-extension test. Green dots indicate points predicted by the optical flow method. Red dots indicate points recognized by the neural network. In (a) and (c), the neural network achieves accurate recognition. By contrast in (b), an error occurs in the coordinates outputted from the deep neural network. If the prediction of the optical flow method can replace the output of the neural network, this error would be corrected.

**Fig. 3.**
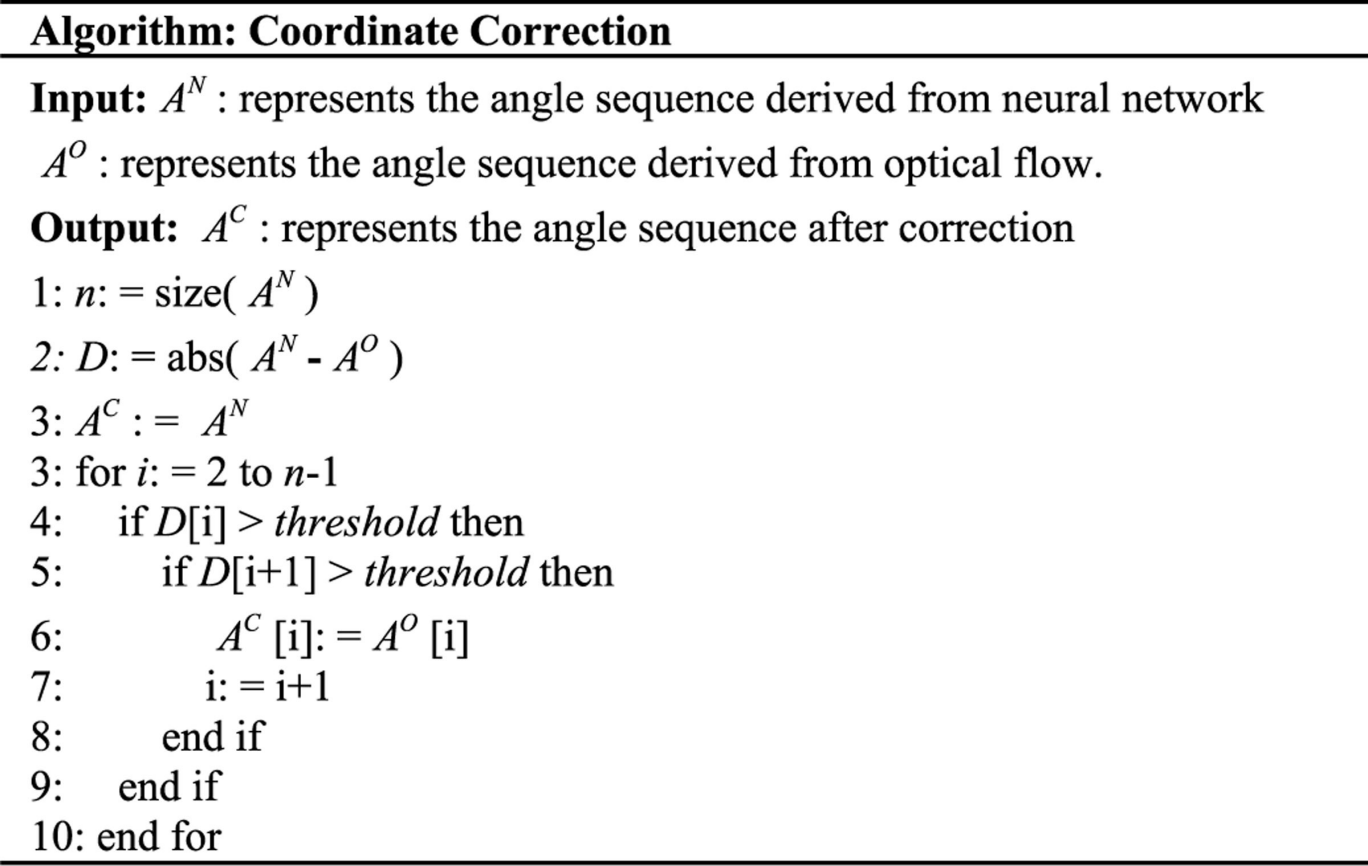
The pseudo codes of the coordinate-correction method.

In the second part, we proposed a coordinate-correction method based on optical flow. Optical flow vectors of the wrist and elbow are calculated by the Lucas-Kanade approach in each frame [17], [25]. Based on our observations on the coordinate set of our neural network, we found that output errors of the neural network normally occurred in a certain frame, in which target keypoint (e.g. elbow, or wrist) turns blurry due to high-speed motion of the upper extremity. This situation is shown in Fig. 2. Therefore, during these frames, we used optical flow method to predict the coordinates set of the elbow and wrist in the next frame based on the coordinate set of these keypoints in the current frame and their corresponding optical flow vectors. The coordinate set predicted by optical flow is used to generate a new angle sequence$A^{O}$. In another aspect, we also cannot always trust the output of optical flow, considering the errors caused by spatial inconsistency or light condition changing. Therefore, we designed an algorithm to correct the wrist and elbow coordinates combining both sequences $A^{N}$ and$A^{O}$, based on a basic assumption that recognition errors of the neural network and optical flow methods do not occur in two consecutive frames at the same time, respectively. The algorithm is displayed in Fig. 3.

### D. Feature Extraction for DTUEPE Metrics

**Fig. 4.**
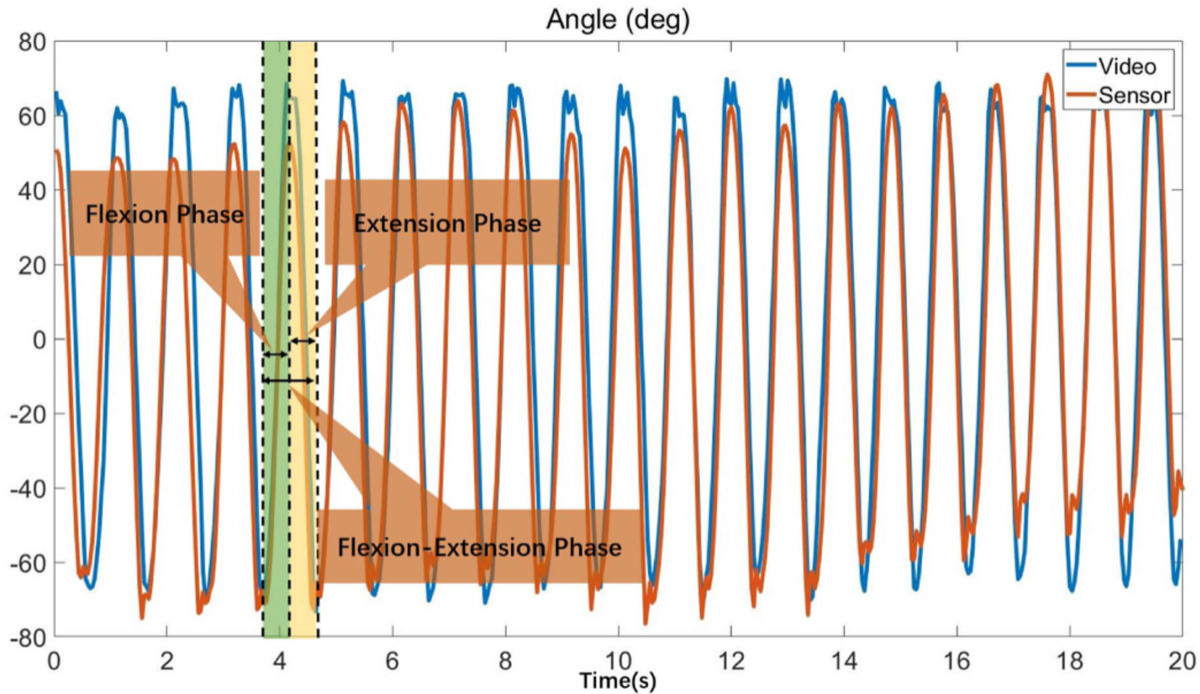
The angle sequences generated by video processing and sensor processing models, where green bar represents extension phase of a period, and yellow bar represents flexion phase. The blue curve represents angle sequences by video processing model, and the orange curve represents angle sequence by sensor processing model.

The mean and standard deviation (SD) of each participants’ extension phase, flexion phase and extension-flexion phase are defined as DTUEPE metrics. Extension and flexion phases for each rotation period are obtained by local peak segmentation method. Flexion phase is defined as the time length of the period when the participant’s elbow flexes. Extension phase is defined as the time length of the period when the participant’s elbow extends. Flexion-extension phase is defined as the time length of a complete period. The method was coded and implemented in MATLAB R2021a (MathWorks, Natick, MA, USA). The segmentation of angle sequence $A^{C}$ is shown as Fig. 4. The local peak segmentation method firstly locates local maximum and minimum peak in angle sequence$A^{C}$. Then, the time from a local maximum to a local minimum is marked as extension phase, and the time from a local minimum to a local maximum is marked as flexion phase.

### E. Clinical Scale

The MMSE test is a 30-point questionnaire that is used extensively in clinical and research settings to measure cognitive impairment [26]. MMSE examines participants’ functions including registration (repeating named prompts), attention and calculation, recall, language, ability to follow simple commands and orientation [27]. The MMSE test usually takes 5 to 10 minutes [27]. The MMSE test is useful for cognitive assessment, although it is biased against people with poor education due to elements of language and mathematical testing, as well as visually impaired, and lack examination of visuospatial cognitive ability. The cognitive statuses of the older adults were measured by MMSE in this work.

The TUG test is a simple test to assess a person’s mobility function and requires his/her static and dynamic balance [18]. In TUG test, participants sit in the chair with their arms on their lap in the beginning. The test begins when participants hear word “Go”. Participants need to rise from the chair, walk three meters, turn around, walk back to the chair, and sit down. The time from the beginning to the completion of the action is TUG scores [18], where its unit is second. The TUG test was used as a measure of physical performance in this work.

### F. Statistical Analyses

To analyze association between DTUEMP metrics derived from the video processing model and the sensor processing model, Spearman correlation coefficient and intraclass correlation coefficient (ICC) were calculated [28]. The cut-off of ICC is that values below 0.50 indicate poor accuracy, values between 0.50 and 0.75 indicate moderate accuracy, values between 0.75 and 0.90 indicate good accuracy, and values above 0.90 indicate excellent accuracy [29]. The cut-off of Spearman correlation coefficient is that values below 0.35 indicate weak correlation, values between 0.35 and 0.67 indicate moderate correlation, and values between 0.68 and 1.0 indicate strong correlation [29].

The main purpose of our model is to create an accessible way that can be used in mobile platforms to help evaluate cognitive and motor functional status. Therefore, we used partial correlation coefficients with control of covariates of age, gender and BMI to study association between video-derived DTUEMP metrics and MMSE scores as well as TUG scores. The cut-off of partial correlation is the same as Spearman correlation coefficient. Statistical analyses were performed using IBM SPSS Statistics version 26 (IBM, Armonk, NY, USA). To meet basic assumption of partial correlation about statistical distribution of dependent variable, the normality of MMSE and TUG score was checked. If the variable does not satisfy normal distribution, fractional rank and inverse distribution function is used to transform the variable to normal distribution before performing correlation analysis. For all statistical analyses, *p* < 0.05 was considered statistically significant.

### G. Multi-Task Learning for DTUEMP to Assess Cognitive and Motor Function

We used MTL framework to analyze the relation between DTUEMP and MMSE as well as TUG scores, with STL framework as benchmark. The z-score of all DTUEMP metrics (both means and SDs) are used as predictors of MTL and STL linear models to predict clinical scales (e.g., MMSE, TUG). In each training fold, *l*_1_-norm regularization with least squares loss was performed to obtain feature sparsity of the models. To validate the effectiveness of the MTL strategy for assessment of cognitive and motor function, we compared mean absolute percentage error (MAPE) derived from MTL and STL linear model with five-fold cross validation [20]. The cut-off of MAPE is the same as David’s work [30]: values below 5% indicate high accuracy, values between 5% and 25% indicate moderate accuracy, and values above 25% indicate unacceptable accuracy.

The reason behind applying multi-task learning is that learning multiple related tasks simultaneously effectively increases the sample size for each task, thereby improving the prediction performance [31]. The MTL model uses least absolute shrinkage and selection operator (Lasso) [32]. The main function is the $l_{1}$−regularized multi-task least squares problem:

$$\underset{W}{\min}\sum_{i=1}^{t}{\left\|W_{i}^{T}X_{i}-Y_{i}\right\|}_{F}^{2}+\rho_{1}{\left\|W\right\|}_{1}+\rho_{L2}{\left\|W\right\|}_{F}^{2}$$

where $X_{i}$ denotes the input matrix of the *i*-th task, $Y_{i}$denotes its corresponding label, $W_{i}$is the model for task *i*, the regularization parameter $\rho_{1}$ controls sparsity, and the optional $\rho_{L2}$ regularization parameter controls the $l_{2}$-nom penalty [31].

### H. Robustness Experiment

In the robustness experiment, we used a common smartphone (model number: HUAWEI Novel 7 Plus) to capture 1080p video of a 20-second elbow flexion-extension test. During the test, participants need to wear a motion sensor (Xsens DOT, Xsens Technologies, Enschede, Netherlands) on the forearm to record the movement. The frame rate of the video is 30 FPS. To verify the robustness of the video processing model under different light conditions and shot angles, we did a series of test with light intensity between 60 and 120 Lux, as well as three different shot angles of 0°, 30°, 60°. The light conditions are divided into three levels: dark, normal, bright. Six young healthy participants were recruited in the robustness experiment to examine the performance of our proposed video processing model on video recordings with different light conditions and shot angles.

## III. Result

### A. Demographics

Twenty older participants who are clinically diagnosed either as mild cognitive impairment (MCI) or dementia are included in this study. Their demographic information is shown in Table I.

### B. Agreement Between DTUEMP Metrics Derived From Video Processing and Sensor Processing Models

The agreement between DTUEMP metrics derived from the video processing model and sensor processing model is shown in Table II. The Spearman correlation coefficients indicated that there was strong correlation with statistical significance for the mean and SD of flexion phase, extension phase and flexion-extension phase between the video processing model and the sensor processing model. The ICC indicated excellent accuracy with statistical significance for the means of flexion phase, extension phase and flexion-extension phase and the SDs of flexion phase and flexion-extension phase, as well as moderate accuracy with statistical significance for the SD of extension phase derived from the video processing model.

### C. Association Between DTUEMP Metrics and Clinical Scale of Cognitive and Motor Function

The normality test of MMSE and TUG scores showed that MMSE was accordance with normal distribution (skewness = − 1.033, kurtosis = − 0.267), while TUG was not in accordance with normal distribution (skewness −2.929, kurtosis = 9.587). Table III shows partial correlation coefficients with control of covariates of age, gender and BMI between DTUEMP metrics measured by video processing model and clinical scale. The means of flexion phase and flexion-extension phase were moderately correlated with MMSE scores with statistical significance (p < 0.05). The SD of flexion phase was strongly correlated with MMSE with statistical significance. The SD of flexion-extension phase was moderately correlated with MMSE with statistical significance. For TUG scores, the means of flexion, extension and flexion-extension phase were moderately correlated with TUG scores with statistical significance.

### D. The Results of Robustness Test

The average age of participants is 22 years, and the mean body mass index (BMI) is 22.56 kg/m^2^. The spearman correlation coefficients and the ICC of robustness test is shown in Table IV. The means of flexion phase, extension phase and flexion-extension phase were strongly correlated with the metrics derived from sensor processing model with statistical significance, and the ICC between most of the means of DTUEMP metrics derived from video processing model and sensor processing model indicated excellent accuracy with statistical significance except flexion phase and extension phase under different shot angles. The means of flexion phase and extension phase derived from the video processing model under different angles had a moderate accuracy with statistical significance according to ICC values. Except the SDs of extension and flexion-extension phase of the test under different light conditions, the SDs of flexion, extension and flexion-extension phases derived from the video processing model had a moderate correlation with those derived from sensor processing model with statistical significance. The correlation between the SDs of extension phase and flexion-extension phase under different light conditions derived from video processing model and sensor processing model indicated strong correlation with statistical significance. All of the SDs of DTUEMP under different light conditions had excellent accuracy according to ICC values, while most of the SDs of DTUEMP (except flexion-extension phase) under different shot angles had good accuracy. The ICC between the SD of flexion-extension phase under different shot angles derived from video processing model and sensor processing model showed moderate accuracy.

### E. The Prediction Performance of MMSE Scores and TUG Scores by DTUEMP Metrics Using Multitask Learning

All of DTUEMP metrics were used as input to MTL and STL linear model. The MAPE of five-fold cross validation of MTL linear model is 17.88% for TUG scores and 18.63% for MMSE scores. The MAPE of five-fold cross validation of STL linear model is 22.53% for TUG scores and 23.18% for MMSE scores. The MAPE of MTL linear model for MMSE and TUG were better than those of STL linear model (benchmark).

## IV. Discussions

In this paper, we proposed a deep learning-based video processing model to extract DTUEMP metrics from a 20-second elbow flexion-extension test to assess cognitive and motor function, thereby potentially assisting the diagnosis of MCR in smartphone platform. As the results indicated, the agreement between DTUEMP measured by video processing model and sensor processing model (benchmark) validated the accuracy of the proposed video processing model. The video-derived DTUEMP metrics were found to be correlated with MMSE and TUG, and could be used to predict MMSE and TUG accurately using MTL linear model. These findings suggested that the DTUEMP metrics can help measure older adults’ cognitive and motor function in a telehealth application.

In Table III, all of the means except the ones of extension phase and flexion-extension phase were found to be correlated with MMSE and TUG scores with statistical significance. In details, all of the DTUEMP metrics are negatively correlated with MMSE scores. As the means of DTUEMP metrics increase, MMSE scores become lower. It means that the increase in each period (i.e. flexion-extension phase) of performing elbow flexion and extension may represent cognitive or motor function decline of the older adults [13]. These findings are consistent previous studies [33], [34] about motor dysfunction of the upper-extremity due to cognitive impairment. The reason behind this association could be explained by previous imaging brain studies in older adults with MCI [35], [36]. In details, brain imagining results showed that inflammatory damage and volumetric changes which are always along with cognitive impairment affected dual-task motor performance (e.g. gait speed). Additionally, the TUG scores are positively correlated with the mean of DTUEMP. The longer extension phase and flexion phase are, the lower athletic ability of the older people have. This finding reflects the consistency of functional mobility between upper and lower extremities. Compared with gait test or TUG test involving lower-extremity motion, upper-extremity functional test is more practical and safer in home environment with limited space. Additionally, from technical view, the camera module housing in a smartphone is easier to capture upper-extremity movement in a fixed location than lower-extremity movements (e.g. walking with a certain distance) within a relatively large space.

In addition to the means of DTUEMP metrics, the SD of DTUEMP metrics were also associated with MMSE negatively and TUG scores positively. A higher SD of DTUEMP represents a weaker functional mobility of the older adult to maintain upper-extremity movements with a stable speed. The associations between SD of DTUEMP and MMSE as well TUG could be explained by different physiological reasons: the older adult with poor motor function (high TUG score) slows down upper-extremity movements gradually due to exhaustion of muscle power in upper extremity at the end of the test, while the older adult with poor cognitive function (low MMSE score) performs upper-extremity movements irregularly due to significant interrupts caused by cognitive task (counting down numbers) during the test. These findings are in line with previous works of our group about physical frailty [37].

In the linear regression analyses, we evaluated model using five-fold cross validation. As our expectation, the MAPE of MTL was better than that of STL. The MAPE of five-fold cross validation showed that the MTL model achieved moderate accuracy for prediction of both MMSE and TUG scores and outperforms the STL model. It is because that a set of related tasks in MTL framework are learnt simultaneously by extracting and utilizing appropriate shared information among tasks [31].

In robust experiment, the Spearman correlation and ICC coefficients between DTUEMP metrics derived from video processing and sensor processing models indicate that our model can work well under different light conditions and shot angles. In details, the correlation of DTUEMP under different shot angles are a little weaker than those under different light conditions. This might be because our neural network is trained mostly based on videos of frontage shot angle in COCO training dataset, and thus is more sensitive to the change of shot angle.

A limitation of this study is that due to the limited number of video recordings we collected in this trial, the neural network used in our video processing model is trained on the COCO *train2017* dataset, which is not specifically designed for tracking upper-extremity movements. Although the used neural network for pose recognition has already achieved sufficient recognition accuracy in our application, the complex model structure of this neural network might restrict the video processing speed when it is deployed in a smartphone platform. Therefore, in future a clinical trial with large sample size and more cohorts of older adults will be conducted to validate the performance of this proposed video processing model. The participants will include not only older adults who are suffering from MCI or dementia, but also with healthy motor and cognitive function. In this way, we can construct a specialized video dataset focusing on upper-extremity movements, and further develop a more accurate deep neural network model for assessing cognitive and motor function by automatically extracting key parameters of upper-extremity motion signals from the video and directly predicting MMSE and TUG scores. Additionally, the model could be more lightweight and computational efficient to be deployed in a smartphone platform without need to upload the video recordings to the cloud for post-processing [38]. The video processing model running in local computation resource of the smartphone can also avoid disclosure of users’ facial information and identification, thereby addressing their privacy concerns.

## V. Conclusion

This paper proposes a video processing model based on a deep residual neural network and optical flow algorithm to measure upper-extremity performance under dual-task condition. The results support safety, acceptability, reliability, and validity of this solution to assess cognitive and motor function from a simple 20-second elbow flexion and extension test. As a summary, this provides another auxiliary diagnostic method of MCR for the older adults in remote areas which can reduce a lot of medical costs. These findings show a promising future of using a simple and cheap tool (i.e. smartphone or tablet housing a video camera owned by older adults or their families) to assess older adults’ cognitive and motor function, thereby monitoring MCR and risk of dementia in telehealth applications.

## Figures and Tables

**TABLE I:** Participants’ Characteristics

Variables	Values

Number of participants	20
Age, years, mean±SD	78.15±6.48
BMI, kg/*m*^2^, mean±SD	25.18±4.07
Female, n (%)	12 (60%)
MMSE, mean±SD	25.75±5.39
TUG, mean±SD	11.42±3.98
MCI, n (%)	15 (75)
Dementia, n (%)	5(25)

BMI: body mass index. MMSE: mini-mental state examination. SD: Standard deviation. TUG: timed up and go test.

**TABLE II:** Agreement Between DTUEMP Metrics From Video Processing Model and Sensor Processing Model

	Correlation coefficients	
	
	$\rho_{s}$	ICC

mean of flexion phase	0.921**	0.979**
SD of flexion phase	0.819**	0.927**
mean of extension phase	0.975**	0.989**
SD of extension phase	0.801**	0.582*
mean of flexion-extension phase	1.000**	0.999**
SD of flexion-extension phase	0.971**	0.971**

*ρ*_*s*_ : Spearman’s correlation coefficients. ICC: interclass correlation coefficients.

* : p < 0.05.

** : p < 0.01. SD: standard deviation

**TABLE III:** Associations Between DTUEMP Metrics and MMSE Scores as Well as TUG Scores

	MMSE		TUG	
		
	r	p	r	p

mean of flexion phase	−0.699	0.005	0.598	0.031
SD of flexion phase	−0.707	0.011	0.362	0.224
mean of extension phase	−0.311	0.280	0.666	0.013
SD of extension phase	−0.054	0.537	0.532	0.061
mean of flexion-extension phase	−0.516	0.059	0.664	0.013
SD of flexion-extension phase	−0.585	0.028	0.419	0.154

MMSE: mini-mental state examination, r: partial correlation coefficients, p: p-value for correlation. SD: standard deviation.

**TABLE IV:** Agreement Between DTUEMP Metrics From Video Processing Model and Sensor Processing Model in Robustness Test

	Mean		Standard deviation	
		
	$\rho_{s}$	ICC	$\rho_{s}$	ICC
				
Flexion (angle)	0.814**	0.651*	0.613*	0.765*
Extension (angle)	0.887**	0.715*	0.634*	0.767*
Flexion-extension (angle)	0.956**	0.974**	0.581*	0.712*
Flexion (light)	0.985**	0.999**	0.635**	0.936**
Extension (light)	0.996**	0.999**	0.684**	0.954**
Flexion-extension (light)	0.991**	1.000**	0.933**	0.994**

* : p < 0.05.

** : p < 0.01.

angle: light condition is 80 lux, with different shot angles, include 0°, 30°, 60°, four participants were involved in each test under different shot angle. light. light: shot angle is 0°, with different light conditions, including dark, mean ± SD = 80.6 ± 1.3416 Lux; normal, mean ± SD = 92.83 ± 4.4008 Lux; bright, mean ± SD = 108.1114 ± 5.5205 Lux.
